# Influence of Menopause on Inflammatory Cytokines during Murine and Human Bone Fracture Healing

**DOI:** 10.3390/ijms19072070

**Published:** 2018-07-16

**Authors:** Verena Fischer, Miriam Kalbitz, Fabian Müller-Graf, Florian Gebhard, Anita Ignatius, Astrid Liedert, Melanie Haffner-Luntzer

**Affiliations:** 1 Institute of Orthopedic Research and Biomechanics, University of Ulm, 89081 Ulm, Germany; verena.fischer@uni-ulm.de (V.F.); fabian@mueller-graf.de (F.M.-G.); anita.ignatius@uni-ulm.de (A.I.); astrid.liedert@uni-ulm.de (A.L.); 2 Center for Trauma Research Ulm (ZTF), University of Ulm, 89081 Ulm, Germany; miriam.kalbitz@uniklinik-ulm.de (M.K.); florian.gebhard@uniklinik-ulm.de (F.G.); 3 Department of Traumatology, Hand-, Plastic-, and Reconstructive Surgery, University Medical Center Ulm, 89081 Ulm, Germany

**Keywords:** midkine, fracture healing, menopause, osteoblastogenesis, bone regeneration, inflammation

## Abstract

Postmenopausal females display a chronic inflammatory phenotype with higher levels of circulating pro-inflammatory cytokines. Furthermore, the inflammatory response to injury may be altered under estrogen-deficiency, because it was shown previously that estrogen-deficient mice displayed increased levels of the inflammatory cytokines Midkine (Mdk) and Interleukin-6 (IL-6) in the early fracture hematoma. Because a balanced immune response to fracture is required for successful bone regeneration, this might contribute to the delayed fracture healing frequently observed in osteoporotic, postmenopausal fracture patients. In this study, we aimed to investigate whether further cytokines in addition to Mdk and IL-6 might be affected by estrogen-deficiency after fracture in mice and whether these cytokines are also relevant during human fracture healing. Additionally, we aimed to investigate whether serum from male vs. female fracture patients affects osteogenic differentiation of human mesenchymal stem cells (MSCs). To address these questions, female mice were either sham-operated or ovariectomized (OVX) and subjected to standardized femur osteotomy. A broad panel of pro- and anti-inflammatory cytokines was determined systemically and locally in the fracture hematoma. In a translational approach, serum was collected from healthy controls and patients with an isolated fracture. Mdk and IL-6 serum levels were determined at day 0, day 14 and day 42 after fracture. Subgroup analysis was performed to investigate differences between male and female fracture patients after menopause. In an in vitro approach, human MSCs were cultured with the collected patient serum and osteogenic differentiation was assessed by qPCR and alkaline-phosphatase staining. Our results suggest an important role for the pro-inflammatory cytokines Mdk and IL-6 in the response to fracture in estrogen-deficient mice among all of the measured inflammatory mediators. Notably, both cytokines were also significantly increased in the serum of patients after fracture. However, only Mdk serum levels differed significantly between male and female fracture patients after menopause. MSCs cultivated with serum from female fracture patients displayed significantly reduced osteogenic differentiation, which was attenuated by Mdk-antibody treatment. In conclusion, our study demonstrated increased Mdk levels after fracture in OVX mice and female fracture patients after menopause. Because Mdk is a negative regulator of bone formation, this might contribute to impaired osteoporotic fracture healing.

## 1. Introduction

Chronic inflammatory conditions, including rheumatoid arthritis, diabetes mellitus and inflammatory bowel disease, which are associated with bone loss, corroborate an intense coupling of the immune and skeletal systems [1,2,3,4]. Postmenopausal osteoporosis is equally considered a chronic inflammatory disease [5]. Postmenopausal osteoporotic females frequently display elevated levels of pro-inflammatory cytokines and alterations in immune cell populations, which were shown to negatively affect bone turnover and quality [6,7,8,9]. Experimental studies in ovariectomized (OVX) rodents, mimicking estrogen decline after menopause, confirmed the pro-inflammatory phenotype, and, moreover, demonstrated an increased inflammatory response to injury, infection and inflammatory conditions [10,11,12,13]. The immune system also plays an important role in bone fracture healing. Notably, the process of bone repair starts with a local inflammatory response at the fracture site [14,15]. This inflammatory reaction is marked by blood vessel disruption, tissue and cell damage and the formation of a fracture hematoma, leading to the recruitment of immune cells and mediators. The cellular composition of the fracture hematoma is initially dominated by polymorphonuclear neutrophils that secrete cytokines and chemokines, including Interleukin-6 (IL-6) and Chemokine (C-X-C motif) ligand-1 (CXCL-1), for further immune-cell recruitment. In addition, the inflammatory response was shown to have an osteogenic potential by recruiting mesenchymal stem cells (MSCs) to the injury site and thus inducing the subsequent repair phase [14]. During the repair phase, intramembranous ossification, which is initiated by periostal osteoblasts and osteoprogenitor cells, and endochondral ossification, which is driven by MSC-derived chondrocytes and later by osteoblasts, guide fracture callus formation. Once the fracture gap is bridged with bone, there is sufficient mechanical stability and the remodeling of the bony fracture callus is initiated [16,17].

Disturbances in this highly dynamic and complex process result in impaired or delayed healing and might contribute to fracture-healing complications frequently observed in postmenopausal, osteoporotic females [18,19]. Experimental studies showed that the depletion of the osteo-anabolic hormone estrogen impaired angiogenesis and delayed endochondral ossification of the fracture callus [20,21,22]. Later during healing, fracture calli of estrogen-deficient rodents displayed a decreased amount of newly formed bone, changes in osteoblast and osteoclast numbers and reduced biomechanical competence [23,24,25,26,27]. These studies indicate that osteoporotic fracture healing is delayed because of impaired angiogenesis and cartilage formation and imbalances in bone cell activities. However, a recent study of our group demonstrated that estrogen-deficiency not only affects the intermediate and late healing stages but also the inflammatory phase after injury [28]. OVX-mice displayed significantly more neutrophils and an increased local expression of the estrogen-responsive and pro-inflammatory cytokine Midkine (Mdk) and IL-6 in the early fracture callus after 3 days. Notably, Mdk-antibody treatment decreased the number of neutrophils and reduced local IL-6 expression in OVX-mice, thus indicating that both Mdk and IL-6 might be involved in the increased presence of neutrophils. Mdk is known as a negative regulator of bone formation, loading-induced bone remodeling and bone repair [29,30,31]. Classic IL-6 signaling was shown to be involved in efficient neutrophil recruitment to the fracture hematoma and to direct endochondral ossification during bone regeneration [32]. Therefore, both cytokines might play an important role during fracture healing. However, numerous other inflammatory mediators, including CXCL-1 and Tumor necrosis factor-α (TNF-α) were shown to influence the fracture-healing process [33,34,35]. Because the expression of many inflammatory cytokines is altered in postmenopausal, osteoporotic patients, as mentioned above, and because a balanced immune response to fracture is required for successful fracture healing, a disturbed and increased inflammatory response to fracture might contribute to the disrupted bone repair of osteoporotic patients.

Therefore, the first aim of this study was to conduct multiplex cytokine analysis in blood and fracture hematoma of sham- and OVX-mice to investigate whether further cytokines in addition to Mdk and IL-6 are affected by estrogen-deficiency. The second aim of this study was to investigate, in a translational approach, whether the regulated cytokines found in sham- vs. OVX-mice are also relevant during human fracture healing and whether their expression differs in male vs. female fracture patients after menopause. Finally, we aimed to investigate whether sera from male vs. female fracture patients affect osteogenic differentiation of human MSCs.

## 2. Results

### 2.1. Inflammatory Response to Fracture in Mice

#### 2.1.1. Increase in Systemic Mdk Concentrations in Estrogen-Deficient Mice after Fracture

**Figure 1:**
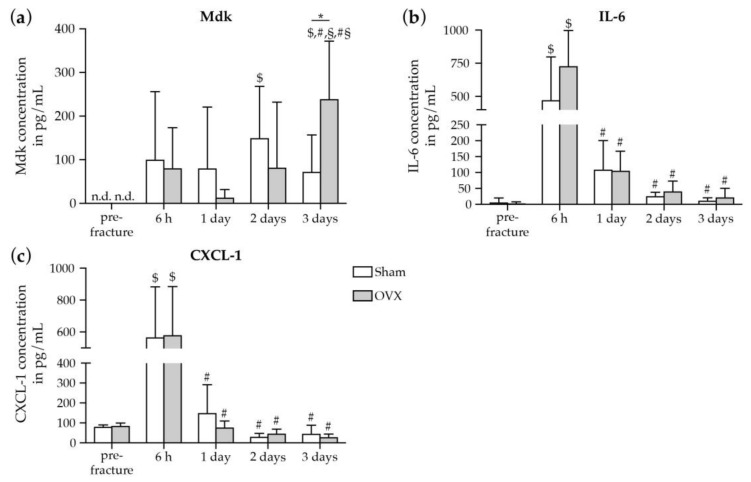
Cytokine/chemokine concentrations in blood plasma of sham- and OVX-mice pre- and after fracture. Plasma levels of (**a**) Mdk, (**b**) IL-6, and (**c**) CXCL-1 in pg/mL. Data represent the mean and standard deviations. Comparison between the groups: *, *p* ≤ 0.05 vs. sham (Student′s *t*-test). Comparison within one group: $, *p* ≤ 0.05 vs pre-fracture, #, *p* ≤ 0.05 vs 6 h, §, *p* ≤ 0.05 vs. 1 day, #§, *p* ≤ 0.05 vs. 2 days (ANOVA with *Post hoc* Fisher′s LSD; *n* = 5–8 per group). n.d. = non-detected, Mdk = Midkine, IL-6 = Interleukin-6, CXCL-1 = chemokine (C-X-C motif) ligand-1.

To assess the impact of estrogen-deficiency on the systemic early immune response after fracture in mice, we measured a broad panel of pro- and anti-inflammatory cytokines and chemokines in the blood plasma of sham- and OVX-mice by multiplex cytokine assay (Figure 1).

Pre-fracture values of measured cytokines did not differ significantly between sham- and OVX-mice. In response to fracture, plasma IL-6 and CXCL-1 levels were significantly increased both in sham- and OVX-mice 6 h after fracture and returned to baseline levels up to 3 days after fracture (Figure 1b,c). However, plasma cytokine and chemokine concentrations did not differ significantly between sham- and OVX-mice at any investigated time point except for the pro-inflammatory and estrogen-responsive cytokine Mdk. In OVX-mice, plasma Mdk concentrations were significantly increased at day 3 after fracture compared to sham-mice (Figure 1a), thus suggesting an increased systemic Mdk release after fracture under estrogen-deficient conditions. IL-6 levels displayed a strong trend towards increased values in OVX-mice at 6 h and 3 days after fracture (Figure 1b). Physiological concentrations of IL-13 and Monocyte chemoattractant protein-1 (MCP-1) were detectable in both groups, however, the concentrations did not increase after fracture and did not change between both groups at the investigated time points (pre-fracture: sham 118 ± 32 vs. OVX 102 ± 70; 6 h: sham 59 ± 56 vs. OVX 97 ± 58; 1 day: sham 50 ± 38 vs. OVX 36 ± 23; 2 days: sham 11 ± 17 vs. OVX 15 ± 21; 3 days: sham 45 ± 47 vs. OVX 14 ± 22 in pg/mL). The additionally measured cytokines and chemokines IL-1β, IL-10, IL-4, TNF-α, Interferon-γ (INF-γ) and Macrophage inflammatory protein-1α (MIP-1α) were not detectable in both groups at any time points.

#### 2.1.2. Increase in Mdk and IL-6 Concentrations in The Fracture Hematoma of Estrogen-Deficient Mice after Fracture

**Figure 2:**
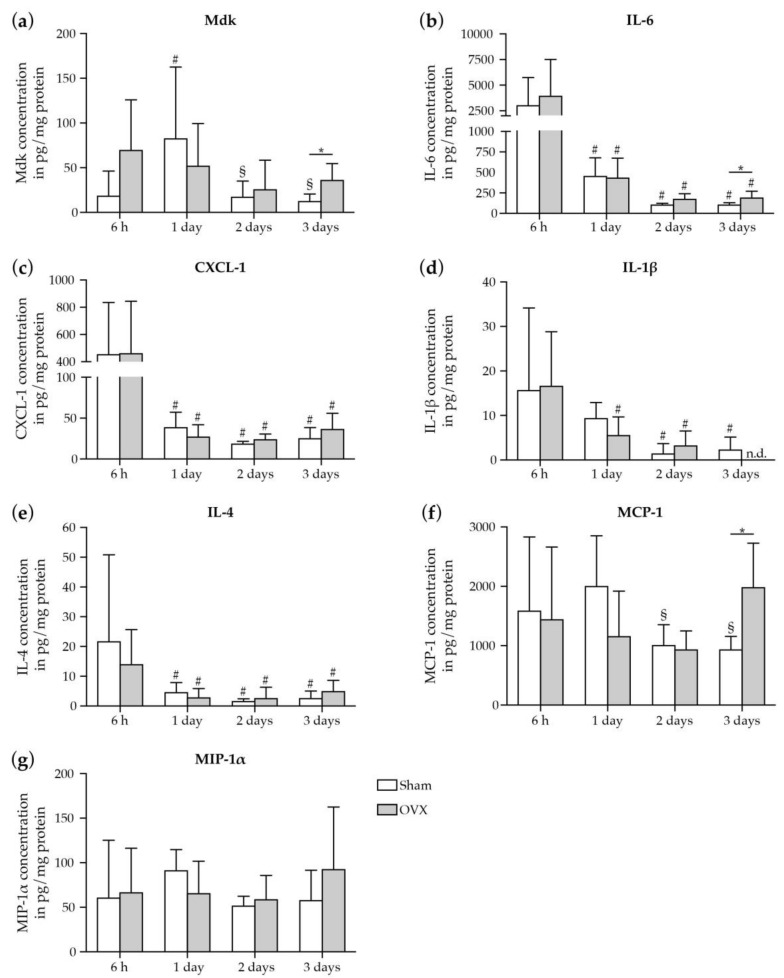
Cytokine/chemokine concentrations in the fracture hematoma of sham- and OVX-mice. Hematoma concentrations of (**a**) Mdk, (**b**) IL-6, (**c**) CXCL-1, (**d**) IL-1β, (**e**) IL-4, (**f**) MCP-1, and (**g**) MIP-1α in pg/mg total protein. Data represent the mean and standard deviations. Comparison between the groups: *, *p* ≤ 0.05 vs. sham (Student′s *t*-test). Comparison within one group: #, *p* ≤ 0.05 vs. 6 h, §, *p* ≤ 0.05 vs. 1 day (ANOVA with *Post hoc* Fisher’s LSD; *n* = 5–8 per group). n.d. = non-detected, Mdk = Midkine, IL-6 = Interleukin-6, CXCL-1 = chemokine (C-X-C motif) ligand-1, IL-1β = Interleukin-1β, IL-4 = Interleukin-4, MCP-1 = monocyte chemoattractant protein-1, MIP-1α = macrophage inflammatory protein-1α.

Next, we investigated the impact of estrogen-deficiency on the local immune response in the murine fracture hematoma (Figure 2).

Locally within the fracture hematoma, we found in both sham- and OVX-mice high concentrations of the pro-inflammatory cytokines IL-6 and CXCL-1 6h post-fracture that progressively declined up to day 3 (Figure 2b,c), which was similarly observed in IL-1β and IL-4 concentrations (Figure 2d,e). Comparing sham- and OVX-mice, Mdk, IL-6, and MCP-1 concentrations were significantly higher in OVX-mice at 3 days after fracture (Figure 2a,b,f), thus suggesting an influence of estrogen-deficiency on the local immune response at the fracture site. No differences in MIP-1α, CXCL-1, IL-1β or IL-4 concentrations were detectable between sham- and OVX-mice (Figure 2c,d,g). IL-10, IL-13, TNF-α and INF-γ were not detectable at the investigated time points in both groups.

### 2.2. Inflammatory Response to Fracture in Humans

#### 2.2.1. Increase in Systemic Mdk Concentrations in Female Fracture Patients after Menopause

**Figure 3:**
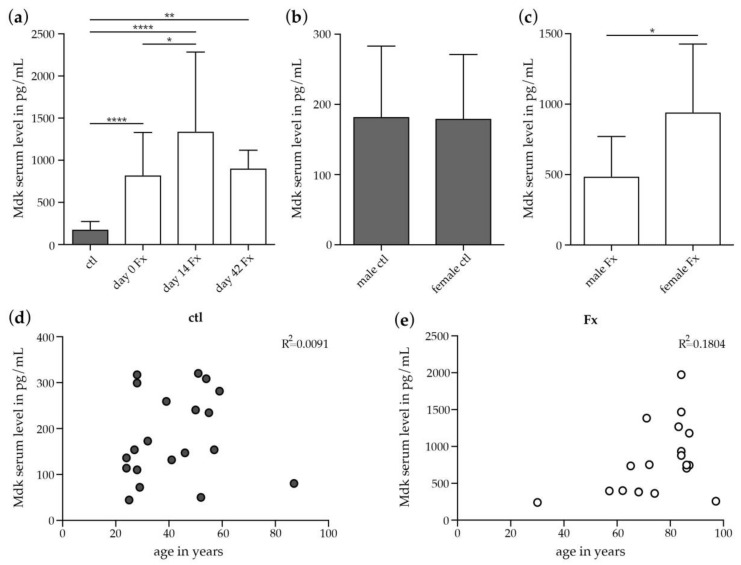
Mdk serum levels in patients with isolated long-bone fracture and healthy controls. (**a**) Mdk serum levels in healthy controls and in fracture patients at day 0, day 14 and day 42 after fracture. Ctl: *n* = 20; d0 Fx: *n* = 26; day 14 Fx: *n* = 7; day 42 Fx: *n* = 4; (**b**) Subgroup analysis of Mdk serum levels in male and female healthy controls. Male ctl: *n* = 10; female ctl: *n* = 10. (**c**) Subgroup analysis of Mdk serum levels in male and female fracture patients after menopause at day 0 after fracture. Male Fx: *n* = 6; female Fx: *n* = 13. (**d**) Pearson’s correlation analysis regarding Mdk serum levels and age in healthy controls. (**e**) Pearson’s correlation analysis regarding Mdk serum levels and age in fracture patients at day 0 after fracture. *, *p* ≤ 0.05, **, *p* ≤ 0.01, ****, *p* ≤ 0.0001, ANOVA with *Post hoc* Fisher’s LSD, Mdk = midkine, ctl = control, Fx = fracture.

Based on the findings observed in mice, we measured in a translational approach Mdk serum concentrations during human fracture healing directly after fracture (day 0), and at days 14 and 42 post-fracture. Mdk serum levels were significantly increased in fracture patients on day 0 compared to healthy controls (Figure 3a). Mdk serum levels were still significantly higher on days 14 and 42 after fracture compared to healthy controls. These results suggest an early rise in Mdk serum levels that remain elevated in response to fracture in patients during the observed time course of healing.

We were next interested in the effects of menopause on Mdk serum levels after fracture. Subgroup analysis revealed that while Mdk serum levels did not differ between male and female healthy controls (Figure 3b), female fracture patients after menopause displayed significantly higher Mdk serum levels on day 0 after fracture compared to male fracture patients (Figure 3c). There was no correlation between Mdk serum levels and age in healthy controls or in fracture patients (Figure 3d,e).

#### 2.2.2. Systemic IL-6 and CRP Concentrations in Female Fracture Patients after Menopause

**Figure 4:**
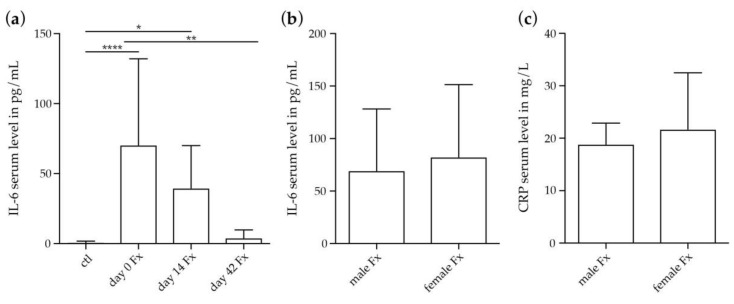
IL-6 and CRP serum levels in patients with isolated long-bone fracture and healthy controls. (**a**) IL-6 serum levels in healthy controls and in fracture patients at day 0, day 14 and day 42 after fracture. Ctl: *n* = 20; day 0 Fx: *n* = 26; day 14 Fx: *n* = 7; day 42 Fx: *n* = 4; (**b**) Subgroup analysis of IL-6 serum levels in male and female fracture patients after menopause at day 0 after fracture. Male Fx: *n* = 6; female Fx: *n* = 13. (**c**) Subgroup analysis of CRP serum levels in male and female fracture patients after menopause at day 0 after fracture. Male Fx: *n* = 6; female Fx: *n* = 13. *, *p* ≤ 0.05, **, *p* ≤ 0.01, ****, *p* ≤ 0.0001, ANOVA with *Post hoc* Fisher’s LSD, IL-6 = Interleukine-6, ctl = control, Fx = fracture, CRP = C-reactive protein.

We further assessed IL-6 serum concentrations in fracture patients post injury and additionally determined C-reactive protein (CRP) serum levels, as a marker of inflammation that is induced following IL-6 secretion. IL-6 was significantly higher in fracture patients at day 0 and day 14 after fracture compared to healthy controls, whereas values returned to baseline at day 42 after fracture (Figure 4a). Subgroups analysis revealed no differences in IL-6 or CRP serum levels in male and female fracture patients after menopause on day 0 (Figure 4b,c).

#### 2.2.3. Effects of Fracture Sera on Osteogenic Differentiation of Human MSCs

**Figure 5:**
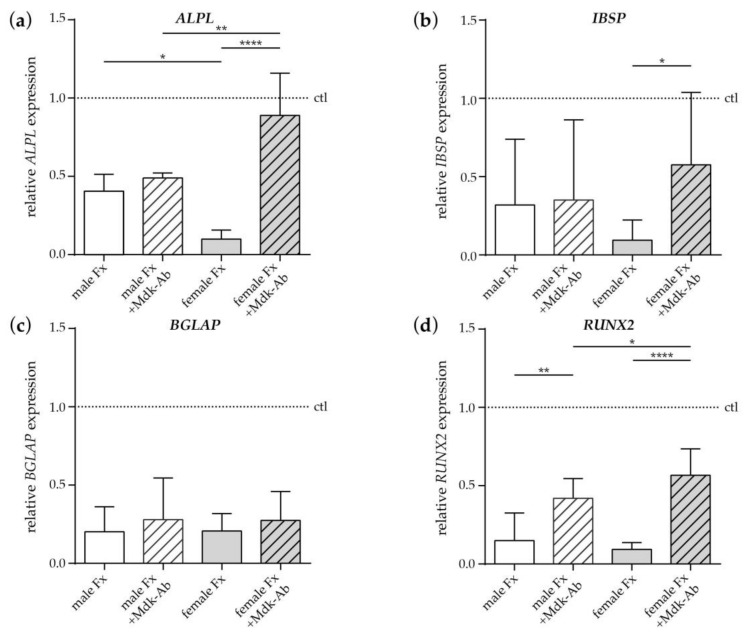

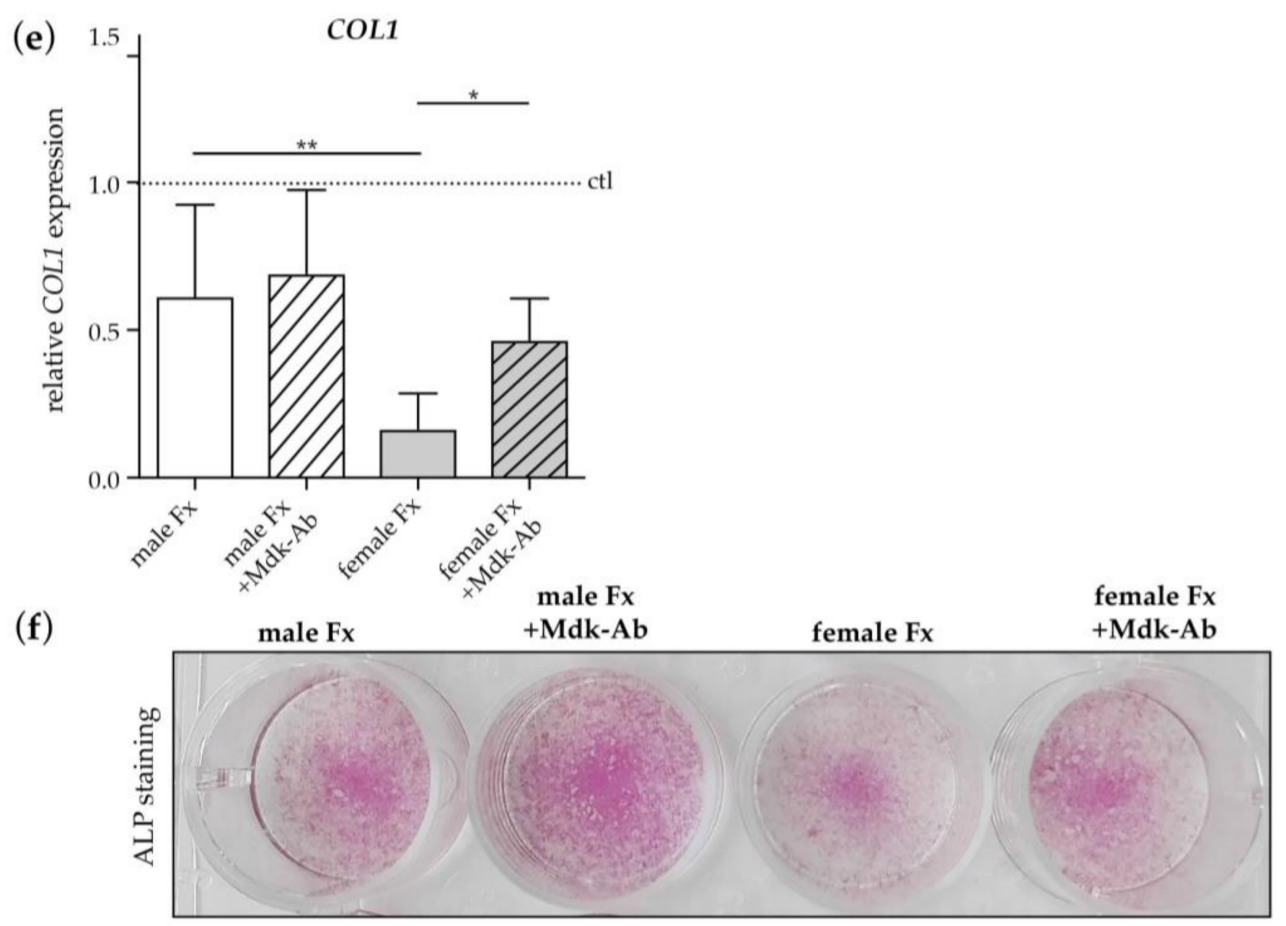
Effects of fracture sera from day 0 after fracture on osteogenic differentiation of human MSCs. (**a**) Relative *ALPL*, (**b**) *ISBP*, (**c**) *BGLAP*, (**d**) *RUNX2* and (**e**) *COL1* gene expression in human MSCs on day 10 of differentiation analyzed by qPCR. Half of the human MSCs were incubated with an inhibitory Midkine antibody (+Mdk-Ab). Values were normalized to GAPDH and the sera of age- and sex-matched healthy controls (dotted line). (**f**) Alkaline phosphatase staining (ALP) of cultured human MSCs on day 10 after treatment with osteogenic medium and sera of male and female fracture patients. Half of cells were incubated with an inhibitory Mdk-Ab. *, *p* ≤ 0.05, **, *p* ≤ 0.01, ****, *p* ≤ 0.0001, ANOVA with *Post hoc* Fisher’s LSD, *n* = 8–10 per group, ctl = control, Fx = fracture, *ALPL* = alkaline phosphatase, *ISPB* = integrin binding sialoprotein, *BGLAP* = bone γ-carboxyglutamate protein, *RUNX2* = runt-related transcription factor 2, *COL1* = collagen-1.

We further assessed the effects of serum from fracture patients and sex-matched healthy controls (dotted line) on the osteogenic differentiation of human MSCs (Figure 5). Fracture serum collected from male and female fracture patients after menopause directly after fracture (day 0) had a negative effect on the expression of the osteogenic marker genes alkaline phosphatase (*ALPL*), integrin-binding sialoprotein (*IBSP*), bone gamma-carboxyglutamate protein (*BGLAP*), Runt-related transcription factor 2 (*RUNX2*) and collagen 1 (*COL1*) (Figure 5a–e). *ALPL* and *COL1* expression were significantly reduced in cells cultivated with female fracture patient serum compared to male fracture patient serum (Figure 5a,e), indicating a more pronounced negative effect of female fracture patient serum. Treatment with an inhibitory Mdk antibody (Mdk-Ab) significantly increased the expression of *RUNX2* in cells cultivated with male fracture patient serum (Figure 5d), whereas the expression of *ALPL*, *IBSP* and *COL1* did not differ (Figure 5a,b,e). In cells cultivated with female fracture patient serum, Mdk-Ab treatment significantly increased the expression of *ALPL*, *IBSP*, *RUNX2* and *COL1*, indicating a more pronounced positive effect of the Ab treatment on the osteogenic differentiation of cells treated with female fracture patient serum. Expression of *BGLAP* did not differ between all treatment groups (Figure 5c). Alkaline phosphatase staining confirmed the more pronounced negative effects of serum from female fracture patients after menopause. However, Mdk-Ab treatment increased alkaline phosphatase staining in cells cultivated with both male and female fracture patient serum, with the highest expression in cells treated with male serum and Mdk-Ab (Figure 5f).

## 3. Discussion

The skeletal and immune systems are closely linked, as shown in postmenopausal osteoporotic females and estrogen-deficient rodents [6,7,8,9,36]. In addition, the immune response to injury is altered under estrogen-deficiency [10,11,13,37,38]. Regarding fracture healing, OVX-mice displayed increased systemic serum Mdk levels on day 10 after fracture [39], increased local Mdk and IL-6 expression and more neutrophils on day 3 in the fracture callus, thus indicating a misbalanced immune response after fracture under estrogen-deficient conditions [28]. Several studies showed that disturbances within the inflammatory phase of fracture healing result in delayed or impaired healing, as demonstrated by depletion of different immune cell types, including neutrophils, macrophages, mast cells and lymphocytes [33,40,41,42,43]. However, an overwhelming immune response also negatively affects bone repair [44,45]. Therefore, the present study should determine whether other cytokines in addition to Mdk and IL-6 might also be affected by estrogen-deficiency. First, we found a fracture-induced increase in IL-6 and CXCL-1 levels that progressively declined up to day 3 after fracture in the plasma of both sham- and OVX-mice, which is in agreement with other experimental studies [32,33]. Confirming our previous study, OVX-mice displayed significantly increased plasma and local callus levels of Mdk at day 3 after fracture. Furthermore, IL-6 increased in the plasma 6 h and 3 days after fracture and significantly increased in the fracture callus 3 days after fracture in OVX- vs. sham-OVX-mice. Notably, all other measured cytokines did not differ in the plasma or callus of sham- or OVX-mice at all the investigated time points, with the exception of the chemoattractant MCP-1, which was significantly increased locally in the fracture callus of OVX-mice at 3 days after fracture. We did not expect the findings of overall no changes in the measured cytokine levels between sham- and OVX-mice, because of the previously mentioned differences in the immune response found in OVX-mice in other injury models displaying altered levels of TNF-α, IL-2, IL-10, IL-1β and IL-6 [10,11,38]. Unexpectedly, also pre-fracture values did not differ significantly between sham- and OVX-mice. By contrast, it was described previously that OVX-mice display altered levels of pro-inflammatory mediators, including IL-6, IL-1β, TNF-α and prostaglandins [12,46]. Both pre-fracture cytokine values and cytokine values in response to fracture might be dependent on the different mouse strains and ages used, on the time span after OVX before analyzing the animals, and on the injury type and severity. Concluding, our previous results and the results of the current study suggest an important role for Mdk and IL-6 in the response to fracture in estrogen-deficient mice. However, other cytokines and chemokines might be involved under estrogen-deficient conditions when the immune system is more challenged, as in case of multiple trauma, hemorrhage or sepsis. It was shown that the application of estrogen in female rodents attenuated the increase of IL-6, TNF-α, IL-1β and MIP-1α after hemorrhage, intestinal injury and thermal injury [47,48,49], thus suggesting more detrimental effects in the case of estrogen-deficiency. This requires further investigation.

Regarding the relevance of Mdk during human fracture healing, previously there have been no studies investigating whether Mdk is present systemically or locally after fracture. Mdk is known to be increased in the serum of patients suffering from colorectal, prostate or lung carcinomas [50,51,52]. Additionally, high blood Mdk levels were demonstrated as a negative predictive factor in neuroblastoma [53] and hepatocellular carcinoma [54]. Furthermore, Mdk was shown to be highly expressed during many inflammatory processes, including diabetic nephropathy [55], atherosclerosis [56], rheumatoid arthritis [57] and sepsis [58]. In the present study, we demonstrated significantly increased Mdk serum levels after isolated long-bone fracture on d0, d14 and d42 after fracture. Because of the involvement of Mdk in many other inflammatory conditions [55,57], and because Mdk was shown to negatively regulate bone formation [29], increased Mdk serum levels after fracture could influence both the early inflammatory phase and the regenerative process after fracture. Interestingly, Mdk serum levels were significantly higher in female fracture patients after menopause, underlying the hypothesis derived from our preclinical data that estrogen-deficiency influences Mdk expression after injury. Indeed, it was shown previously that the promoter region of the *Mdk* gene contains estrogen-responsive elements [59]. However, in contrast to our preclinical data, IL-6 serum levels did not differ between male and female fracture patients after menopause, indicating that the effect of estrogen-deficiency is less pronounced on this cytokine in humans. Furthermore, unchanged CRP serum levels in males vs. females might indicate no further changes in the general immune status in our fracture patients. However, in fracture patients we did not investigate the entire panel of inflammatory mediators that we measured in the preclinical study, because we focused on the results obtained in the preclinical study. Therefore, we cannot currently exclude the possibility that other inflammatory mediators might be affected in response to fracture in patients, which needs further investigation. Nonetheless, our preclinical and clinical data suggest an important role for Mdk, particularly during estrogen-deficient conditions, in response to fracture.

Because Mdk was shown to negatively affect osteogenic differentiation based on an inhibition of the osteo-anabolic Wnt/β-catenin pathway [29,31], we next investigated in an in vitro approach whether the serum of fracture patients, in which we found increased Mdk serum levels, might influence osteogenic differentiation of human MSCs. Fracture serum from both males and females after menopause negatively affected osteogenic differentiation of human MSCs. In previous studies, a negative effect of human fracture-patient serum directly and up to one week after fracture was demonstrated on the proliferation of osteogenic SaOS-2 cells, a human osteosarcoma cell line, and human MSCs [60,61]. This might result from declined levels of insulin-like growth factor-1 and transforming growth factor-β during the first 3 days after long-bone fracture, which was found in another study [62]. However, no correlation was found between the levels of circulating growth factors and age or sex of the fracture patient [62]. In the present study, osteogenic differentiation of human MSCs was reduced even further in cells cultivated with the serum obtained from female fracture patients after menopause compared to male fracture serum. In addition, treatment with Mdk-Ab increased the osteogenic differentiation potential more pronouncedly in cells treated with serum from female fracture patients compared to male fracture patients, since both mRNA and protein level of alkaline phosphatase was increased after Mdk-Ab treatment in cells treated with female fracture serum, whereas only protein expression of alkaline phosphatase was increased after Mdk-Ab treatment in cells treated with male fracture serum. This might be also due to the fact that we analyzed mRNA and protein expression at the same time point during osteogenic differentiation. Together with the finding that Mdk serum levels are increased in the female patient cohort, we propose that Mdk might be an important signaling molecule in the response to fracture, particularly under estrogen-deficient conditions. Because we demonstrated previously that Mdk-Ab treatment significantly accelerated fracture healing in osteoporotic, OVX-mice [39], this indicates a therapeutic potential for the Mdk-Ab during delayed fracture healing in postmenopausal osteoporotic female patients.

Our study has several limitations. First, we did not have access to serum from premenopausal female fracture patients to compare with serum from female fracture patients after menopause, which would have provided a better control for estrogen-deficiency than male fracture patients. Second, we cannot be absolutely certain whether the female fracture patients were post-menopausal, because we did not specifically determine this. However, based on the age ranging from 57 to 87, with a mean age of 79, we strongly assume that the female fracture patients of the present study were menopausal. Third, our healthy controls were significantly younger than our fracture patients. However, we did not detect a significant correlation between Mdk serum levels and age in both healthy controls and fracture patients. In addition, we were not able to obtain serum from more fracture patients at later time points to perform a subgroup analysis between males and females after menopause also at day 14 and day 42. This would be an interesting investigation for future studies. Furthermore, it would be highly relevant to investigate the local Mdk expression in the human fracture hematoma or callus. Finally, we did not evaluate fracture healing outcome in fracture patients. It would be interesting to investigate in the future whether changes in Mdk serum levels do in fact correlate with the occurrence of fracture-healing complications and healing outcome.

## 4. Materials and Methods

### 4.1. Evaluation of The Inflammatory Response to Fracture in Mice

All experiments were performed in accordance with the international regulations for the care and use of laboratory animals (Directive 2010/63/EU) and with the approval of the local ethical committee (Regierungspräsidium Tübingen, Germany, No. 1184, approval date: 4 July 2014).

#### 4.1.1. Experimental Study Design

Female C57BL/6J mice (*n* = 47; 5–8 mice per group) were purchased from Charles River (Sulzfeld, Germany) and maintained in groups of two to five animals per cage (370 cm^2^) under a 14 h light and 10 h dark circadian rhythm. Mice received a standard mouse food or a phytoestrogen-free food for OVX-mice (ssniff R/M-H, V1535 or V1554, Sniff, Soest, Germany) and water *ad libitum*. When aged 3–4 months, half of the mice underwent bilateral OVX to induce estrogen-deficiency, whereas the remaining half was sham-operated. Femur osteotomy was performed 8 weeks after sham/OVX operation. Pre-fracture blood samples (sham: 6 mice, OVX: 5 mice) were taken from mice under general anesthesia by punctuation of the *vena facialis*. Mice were euthanized 6 h (sham: 5 mice, OVX: 7 mice), 1 day (sham: 8 mice, OVX: 6 mice), 2 days (sham: 5 mice, OVX: 5 mice) or 3 days (sham: 6 mice, OVX: 5 mice) after osteotomy, and the effects of estrogen-deficiency on the early immune response after fracture were evaluated.

#### 4.1.2. Ovariectomy and Osteotomy Surgeries

OVX and femur osteotomy were performed under general anesthesia with 2% isoflurane. For analgesia and antibiosis, mice received 25 mg/L tramadol hydrochloride (Tramal, Gruenenthal, Aachen, Germany) via the drinking water (1 day prior and 3 days post-surgery) and a single subcutaneous injection of the antibiotic clindamycin-2-dihydrogenphosphate (45 mg/kg, Clindamycin, Ratiopharm, Ulm, Germany) prior to surgery, respectively. Mice were bilaterally ovariectomized by ligation of the oviduct and removal of the ovary as described previously [28]. After 8 weeks, femur osteotomy was performed as described previously [39]. Briefly, an osteotomy gap was created using a 0.4 mm Gigli saw (RISystem, Davos, Switzerland) at the mid-shaft of the diaphysis of the right femur and stabilized using an external fixator (axial stiffness 3 N/mm, RISystem).

#### 4.1.3. Cytokine and Chemokine Analyses in Blood Plasma and Fracture Hematoma

To investigate the effects of estrogen-deficiency on the systemic and local early immune responses after fracture (6 h: sham 5 and OVX 7 mice; 1 day: sham 8 and OVX 6 mice; 2 days: sham 5 and OVX 5 mice; 3 days: sham 6 and OVX 5 mice), we assessed cytokine and chemokine concentrations in blood plasma and the fracture hematoma by multiplex immunoassay and enzyme-linked immunosorbent assay (ELISA). In addition, pre-fracture blood samples were taken by punctuation of the *vena facialis* from 6 sham and 5 OVX mice directly before surgery. Cardiac blood was collected in plasma microvettes and centrifuged at 14,000 rpm and 4 °C for 10 min. The harvested fracture hematoma was lysed in lysis buffer containing protease inhibitors (10 mM Tris pH 7.5, 10 mM NaCl, 0.1 mM EDTA, 0.5% Triton-X 100, 0.02% NaN_3_, 0.2 mM phenylmethylsulfonyl fluoride; and Halt Protease and Phosphate Inhibitor Single-Use Cocktail, Thermo Fisher Scientific, Waltham, MA, USA). Total fracture hematoma protein concentration was determined using the Pierce BCA Protein Assay Kit (Thermo Fisher Scientific). Using a customized mouse Multiplex Cytokine Kit (ProcartaPlex; eBioscience, Frankfurt, Germany), plasma and fracture hematoma concentrations of IL-6, IL-1β, IL-13, IL-10, IL-4, CXCL-1, MCP-1, MIP-1α, INF-γ and TNF-α were determined. The plasma and fracture hematoma levels of Mdk were determined using Midkine ELISA (Cellmid Limited, Sydney, Australia), according to the manufacturer’s protocol.

### 4.2. Evaluation of The Inflammatory Response to Fracture in Humans

#### 4.2.1. Clinical Study Design

This study was approved by the Ethical Committee of the University Medical Center Ulm and conducted in accordance with the declaration of Helsinki. In total, 26 patients (age: 32–97 years, mean: 75 years) with metaphyseal/diaphyseal fractures of long bones (femur, tibia, humerus, radius, ulna) treated surgically at the University Medical Center Ulm between January 2016 and January 2018 were included. All patients gave written consent to be enrolled in the study. Exclusion criteria were polytrauma, pregnancy, bone diseases except primary osteoporosis, intake of bisphosphonates or parathyroid hormone, rheumatoid arthritis, open fractures grade 3 or 4 according to Tscherne and Oestern, hepatic or nephritic insufficiency, cancer, intake of steroids, intake of immunosuppressive medication, chemotherapy in the last 3 months and artificial ventilation after surgery. In further subgroup analyses, patients with femur fracture (AO-31 A1/A2/A3/B2; *n* = 19) were assigned to two groups at d0: male fracture patients (*n* = 6, age: 32–97 years, mean: 69 years) and female fracture patients after menopause (*n* = 13, age: 57–87 years, mean: 78 years). Furthermore, 20 healthy volunteers (10 males aged 24–57, mean: 37; 10 females aged 27–87 years, mean: 47 years) donated one blood sample each as controls.

#### 4.2.2. Blood Samples

Peripheral venous blood was obtained from each patient (*n* = 26) at 1–24 h after the fracture event (day 0). From seven patients, peripheral venous blood was also obtained 14 ± 1 days after the fracture event (day 14). Furthermore, from four patients, peripheral venous blood was obtained 42 ± 2 days after the fracture event (day 42). Blood samples were centrifuged to obtain serum and stored at −80 °C until analysis. CRP was analyzed using a routine clinical chemistry analyzer, Dimension RxL (Dade Behring) in accordance with the manufacturer’s protocol. A value below 5 mg/L is considered to be normal.

#### 4.2.3. ELISAs

Mdk (Cellmid Ltd) and IL-6 (Quantikine, R&D Systems, Minneapolis, MN, USA) ELISAs were performed according to the manufacturers’ instructions. Samples were determined in duplicate. To avoid interassay variability influencing the results, samples from fracture patients and control subjects were randomly assigned to the ELISA plates.

### 4.3. Evaluation of The Effects of Fracture Sera on Human MSCs In Vitro

#### 4.3.1. Cell-Culture Experiments

Human bone marrow-derived MSCs were obtained from Lonza (Basel, Switzerland). Cells were cultivated in α-MEM supplemented with 10% human serum, 1% penicillin/streptomycin and 1% l-glutamine (all Thermo Fisher Scientific) at 37 °C under 5% CO_2_. Cells were seeded at 10,000 cells per well in 24-well plates. For induction of osteogenic differentiation, medium was supplemented with 10 mM β-glycerophosphate and 0.2 mM ascorbate. Osteogenic differentiation was conducted for 10 days. As experimental groups, cells were cultured with pooled serum from 3–4 male control subjects/female control subjects/male fracture patients day 0/female fracture patients after menopause day 0. Mdk-Ab was applied as described previously [31]. Subsequently, cells were fixed and stained for alkaline phosphatase as described previously [63] or total RNA was isolated. Experiments were performed in duplicates or triplicates at least three times.

#### 4.3.2. qPCR

Cells were lysed in commercially available RLT lysis buffer (Qiagen, Hilden, Germany) containing 10 μL/mL β-mercaptoethanol (Merck, Darmstadt, Germany). Lysates were homogenized with QIAshredder columns and total RNA was isolated using the RNeasy Mini kit (both Qiagen). DNA digestion was performed using the RNase-free DNase kit (Qiagen). All steps were conducted according to the manufacturer′s protocols. For real-time PCR, the SensiFAST™ SYBR^®^ Hi-ROX One-Step Kit (Bioline, Luckenwalde, Germany) was used according to the manufacturer’s instructions. The PCR protocol was 45 °C for 10 min, 95 °C for 2 min, 40 cycles with 95 °C for 5 s, 60 °C for 15 s and 72 °C for 15 s. For melting-curve analysis, the procedure was 60 °C for 1 min and heating in 0.15 °C steps up to 95 °C. The relative amount of RNA was calculated using the ΔΔCt (cycle threshold) method: The Ct values from the fracture patient samples were normalized to the mean Ct values of the control subject samples of the same sex (ΔCt) and the reference gene (ΔΔCt, Gapdh). The relative mRNA expression was calculated by the term: $\frac{\mathrm{PCR}\;\mathrm{Efficiency}\;\left(\mathrm{gene}\;\mathrm{of}\;\mathrm{interest}\right){\;}^{\Delta \mathrm{Ct}\;\left(\mathrm{gene}\;\mathrm{of}\;\mathrm{interest}\right)}}{\mathrm{PCR}\;\mathrm{Efficiency}\;{\left(\mathrm{reference}\;\mathrm{gene}\right)}^{\Delta \mathrm{Ct}\;\left(\mathrm{reference}\;\mathrm{gene}\right)}}$. The PCR efficiencies of the different primer pairs were calculated using the software LinRegPCR as published previously [64]. Primer sequences were as follows: *ALPL* for: 5′-CCTCGGAAGACACTCTGACC-3′, rev: 5′-CCACCAAATGTGAAGACGTG-3′; *IBSP* for: 5′-CGAGGGGGAGTACGAATACA-3′, rev: 5′-AGGTTCCCCGTTCTCACTTT-3′; *RUNX2b* for: 5′-GCAGTTCCCAAGCATTTCAT-3′, rev: 5′-CACTCTGGCTTTGGGAAGAG-3′; *BGLAP* for: 5′-GGCAGCGAGGTAGTGAAGAG-3′, rev: 5′-CTCCCAGCCATTGATACAGG-3′; *COL1* for: 5′-TGACCTCAAGATGTGCCACT-3′, rev: 5′-ACCAGACATCCCTCTTGTCC-3′; *GAPDH* for: 5′-GCGACAACATCCAGGGTATC-3′, rev: 5′-GAAGATGGTGATGGGATTTC-3′.

### 4.4. Statistical Analysis

Results are presented as the mean and standard deviation. Statistical analysis was performed using GraphPad Prism 7 software (GrapdPad Software, Inc., La Jolla, CA, USA). Normal distribution of all data was tested by the Shapiro-Wilk normality test. Experimental in vivo data were analyzed for significance either by Student’s *t*-test when sham and OVX groups were compared or by one-way analysis of variance (ANOVA) with post-hoc Fischer’s LSD when three or more time points were compared. The results of the clinical study were analyzed with Student′s *t*-test or ANOVA with post-hoc Fisher’s LSD dependent on the number of analyzed groups. Correlations between age and Mdk serum levels were identified by Pearson’s correlation analysis. The level of significance was set at *p* ≤ 0.05. The number of samples for each experiment is indicated in the figure legends and tables.

## 5. Conclusions

In conclusion, our study demonstrated increased Mdk levels after fracture in OVX mice and female fracture patients after menopause. Because Mdk is a negative regulator of bone formation, this might contribute to impaired osteoporotic fracture healing. Confirming this, our in vitro data showed increased osteogenic differentiation of human MSCs treated with serum of female fracture patients after Mdk-Ab treatment, indicating that Mdk might be a target to particularly improve osteoporotic bone repair in female fracture patients after menopause.

## 6. Patents

Jones, D.; Halasz, M.; Liedert, A.; Ignatius, A.; Haffner-Luntzer, M. Methods of treating bone diseases, disorders and/or injury and reagents thereof. Cellmid Limited, Australian Patent in process.

## Abbreviations

ANOVA	Analysis of variance
ALPL	Alkaline phosphatase
BCA	Bicinchoninic acid
BGLAP	Bone gamma-carboxyglutamate protein
COL1	Collagen-1
CRP	C-reactive protein
Ctl	Control
CXCL-1	Chemokine (C-X-C motif) ligand-1
ELISA	Enzyme-linked immunosorbent assay
Fx	Fracture
GAPDH	Glyceraldehyde-3-phosphate dehydrogenase
IBSP	Integrin-binding sialoprotein
IL	Interleukin
MCP-1	Monocyte chemoattractant protein-1
Mdk	Midkine
Mdk-Ab	Midkine-antibody
MIP-1α	Macrophage inflammatory protein-1α
MSC	Mesenchymal stem cell
ND	Non-detected
OVX	Ovariectomized
qPCR	Quantitative polymerase chain reaction
RNA	Ribonucleic acid
RUNX2	Runt-related transcription factor 2
TNF-α	Tumor necrosis factor-α

## References

[1] Walsh N.C., Gravallese E.M. (2004). Bone loss in inflammatory arthritis: Mechanisms and treatment strategies. Curr. Opin. Rheumatol..

[2] Jensen T., Klarlund M., Hansen M., Jensen K.E., Skjodt H., Hyldstrup L., Danisit T.G. (2004). Connective tissue metabolism in patients with unclassified polyarthritis and early rheumatoid arthritis. Relationship to disease activity, bone mineral density, and radiographic outcome. J. Rheumatol..

[3] Wu Y.Y., Xiao E., Graves D.T. (2015). Diabetes mellitus related bone metabolism and periodontal disease. Int. J. Oral. Sci..

[4] Moschen A.R., Kaser A., Enrich B., Ludwiczek O., Gabriel M., Obrist P., Wolf A.M., Tilg H. (2005). The RANKL/OPG system is activated in inflammatory bowel disease and relates to the state of bone loss. Gut.

[5] Ginaldi L., Di Benedetto M.C., De Martinis M. (2005). Osteoporosis, inflammation and ageing. Immun. Ageing.

[6] Ginaldi L., De Martinis M., Ciccarelli F., Saitta S., Imbesi S., Mannucci C., Gangemi S. (2015). Increased levels of interleukin 31 (IL-31) in osteoporosis. BMC Immunol..

[7] Pfeilschifter J., Koditz R., Pfohl M., Schatz H. (2002). Changes in proinflammatory cytokine activity after menopause. Endocr. Rev..

[8] Weitzmann M.N., Pacifici R. (2006). Estrogen regulation of immune cell bone interactions. Ann. N. Y. Acad. Sci..

[9] Pacifici R. (2008). Estrogen deficiency T cells and bone loss. Cell. Immunol..

[10] Shivers K.Y., Amador N., Abrams L., Hunter D., Jenab S., Quinones-Jenab V. (2015). Estrogen alters baseline and inflammatory-induced cytokine levels independent from hypothalamic-pituitary-adrenal axis activity. Cytokine.

[11] Routley C.E., Ashcroft G.S. (2009). Effect of estrogen and progesterone on macrophage activation during wound healing. Wound Repair Regen..

[12] Stubelius A., Andersson A., Islander U., Carlsten H. (2017). Ovarian hormones in innate inflammation. Immunobiology.

[13] Ashcroft G.S., Dodsworth J., van Boxtel E., Tarnuzzer R.W., Horan M.A., Schultz G.S., Ferguson M.W. (1997). Estrogen accelerates cutaneous wound healing associated with an increase in TGF-beta1 levels. Nat. Med..

[14] Claes L., Recknagel S., Ignatius A. (2012). Fracture healing under healthy and inflammatory conditions. Nat. Rev. Rheumatol..

[15] Walters G., Pountos I., Giannoudis P.V. (2018). The cytokines and micro-environment of fracture haematoma: Current evidence. J. Tissue Eng. Regen. Med..

[16] Einhorn T.A., Gerstenfeld L.C. (2015). Fracture healing: Mechanisms and interventions. Nat. Rev. Rheumatol..

[17] Einhorn T.A. (1998). The cell and molecular biology of fracture healing. Clin. Orthop. Relat. Res..

[18] Nikolaou V.S., Efstathopoulos N., Kontakis G., Kanakaris N.K., Giannoudis P.V. (2009). The influence of osteoporosis in femoral fracture healing time. Injury.

[19] Cheung W.H., Miclau T., Chow S.K., Yang F.F., Alt V. (2016). Fracture healing in osteoporotic bone. Injury.

[20] Beil F.T., Barvencik F., Gebauer M., Seitz S., Rueger J.M., Ignatius A., Pogoda P., Schinke T., Amling M. (2010). Effects of estrogen on fracture healing in mice. J. Trauma Acute Care Surg..

[21] Xu S.W., Yu R., Zhao G.F., Wang J.W. (2003). Early period of fracture healing in ovariectomized rats. Chin. J. Traumatol..

[22] Hatano H., Siegel H.J., Yamagiwa H., Bronk J.T., Turner R.T., Bolander M.E., Sarkar G. (2004). Identification of estrogen-regulated genes during fracture healing, using DNA microarray. J. Bone Miner. Metab..

[23] Namkung-Matthai H., Appleyard R., Jansen J., Hao Lin J., Maastricht S., Swain M., Mason R.S., Murrell G.A., Diwan A.D., Diamond T. (2001). Osteoporosis influences the early period of fracture healing in a rat osteoporotic model. Bone.

[24] Meyer R.A., Tsahakis P.J., Martin D.F., Banks D.M., Harrow M.E., Kiebzak G.M. (2001). Age and ovariectomy impair both the normalization of mechanical properties and the accretion of mineral by the fracture callus in rats. J. Orthop. Res..

[25] Wang J.W., Li W., Xu S.W., Yang D.S., Wang Y., Lin M., Zhao G.F. (2005). Osteoporosis influences the middle and late periods of fracture healing in a rat osteoporotic model. Chin. J. Traumatol.

[26] Hao Y.J., Zhang G., Wang Y.S., Qin L., Hung W.Y., Leung K., Pei F.X. (2007). Changes of microstructure and mineralized tissue in the middle and late phase of osteoporotic fracture healing in rats. Bone.

[27] Islam A.A., Rasubala L., Yoshikawa H., Shiratsuchi Y., Ohishi M. (2005). Healing of fractures in osteoporotic rat mandible shown by the expression of bone morphogenetic protein-2 and tumour necrosis factor-alpha. Br. J. Oral Maxillofac. Surg..

[28] Haffner-Luntzer M., Fischer V., Prystaz K., Liedert A., Ignatius A. (2017). The inflammatory phase of fracture healing is influenced by oestrogen status in mice. Eur. J. Med. Res..

[29] Liedert A., Mattausch L., Rontgen V., Blakytny R., Vogele D., Pahl M., Bindl R., Neunaber C., Schinke T., Harroch S. (2011). Midkine-deficiency increases the anabolic response of cortical bone to mechanical loading. Bone.

[30] Neunaber C., Catala-Lehnen P., Beil F.T., Marshall R.P., Kanbach V., Baranowsky A., Lehmann W., Streichert T., Ignatius A., Muramatsu T. (2010). Increased trabecular bone formation in mice lacking the growth factor midkine. J. Bone Miner. Res..

[31] Haffner-Luntzer M., Heilmann A., Rapp A.E., Roessler R., Schinke T., Amling M., Ignatius A., Liedert A. (2016). Antagonizing midkine accelerates fracture healing in mice by enhanced bone formation in the fracture callus. Br. J. Pharmacol..

[32] Prystaz K., Kaiser K., Kovtun A., Haffner-Luntzer M., Fischer V., Rapp A.E., Liedert A., Strauss G., Waetzig G.H., Rose-John S. (2018). Distinct Effects of IL-6 Classic and Trans-Signaling in Bone Fracture Healing. Am. J. Pathol..

[33] Kovtun A., Bergdolt S., Wiegner R., Radermacher P., Huber-Lang M., Ignatius A. (2016). The crucial role of neutrophil granulocytes in bone fracture healing. Eur. Cells Mater..

[34] Kayal R.A., Siqueira M., Alblowi J., McLean J., Krothapalli N., Faibish D., Einhorn T.A., Gerstenfeld L.C., Graves D.T. (2010). TNF-alpha mediates diabetes-enhanced chondrocyte apoptosis during fracture healing and stimulates chondrocyte apoptosis through FOXO1. J. Bone Miner. Res..

[35] Chan J.K., Glass G.E., Ersek A., Freidin A., Williams G.A., Gowers K., Espirito Santo A.I., Jeffery R., Otto W.R., Poulsom R. (2015). Low-dose TNF augments fracture healing in normal and osteoporotic bone by up-regulating the innate immune response. EMBO Mol. Med..

[36] Hardy R., Cooper M.S. (2009). Bone loss in inflammatory disorders. J. Endocrinol..

[37] Aydin A., Halici Z., Albayrak A., Polat B., Karakus E., Yildirim O.S., Bayir Y., Cadirci E., Ayan A.K., Aksakal A.M. (2015). Treatment with Carnitine Enhances Bone Fracture Healing under Osteoporotic and/or Inflammatory Conditions. Basic Clin. Pharmacol. Toxicol..

[38] Inoue K., Inoue E., Imai Y. (2013). Female sex hormones ameliorate arthritis in SKG mice. Biochem. Biophys. Res. Commun..

[39] Haffner-Luntzer M., Kemmler J., Heidler V., Prystaz K., Schinke T., Amling M., Kovtun A., Rapp A.E., Ignatius A., Liedert A. (2016). Inhibition of Midkine Augments Osteoporotic Fracture Healing. PLoS ONE.

[40] Kroner J., Kovtun A., Kemmler J., Messmann J.J., Strauss G., Seitz S., Schinke T., Amling M., Kotrba J., Froebel J. (2017). Mast Cells Are Critical Regulators of Bone Fracture-Induced Inflammation and Osteoclast Formation and Activity. J. Bone Miner. Res..

[41] Alexander K.A., Chang M.K., Maylin E.R., Kohler T., Muller R., Wu A.C., Van Rooijen N., Sweet M.J., Hume D.A., Raggatt L.J. (2011). Osteal macrophages promote in vivo intramembranous bone healing in a mouse tibial injury model. J. Bone Miner. Res..

[42] Sun G., Wang Y., Ti Y., Wang J., Zhao J., Qian H. (2017). Regulatory B cell is critical in bone union process through suppressing proinflammatory cytokines and stimulating Foxp3 in Treg cells. Clin. Exp. Pharmacol. Physiol..

[43] Reinke S., Geissler S., Taylor W.R., Schmidt-Bleek K., Juelke K., Schwachmeyer V., Dahne M., Hartwig T., Akyuz L., Meisel C. (2013). Terminally differentiated CD8(+) T cells negatively affect bone regeneration in humans. Sci. Transl. Med..

[44] Kemmler J., Bindl R., McCook O., Wagner F., Groger M., Wagner K., Scheuerle A., Radermacher P., Ignatius A. (2015). Exposure to 100% Oxygen Abolishes the Impairment of Fracture Healing after Thoracic Trauma. PLoS ONE.

[45] Recknagel S., Bindl R., Kurz J., Wehner T., Ehrnthaller C., Knoferl M.W., Gebhard F., Huber-Lang M., Claes L., Ignatius A. (2011). Experimental blunt chest trauma impairs fracture healing in rats. J. Orthop. Res..

[46] Polat B., Halici Z., Cadirci E., Albayrak A., Karakus E., Bayir Y., Bilen H., Sahin A., Yuksel T.N. (2013). The effect of alpha-lipoic acid in ovariectomy and inflammation-mediated osteoporosis on the skeletal status of rat bone. Eur. J. Pharmacol..

[47] Yokoyama Y., Toth B., Kitchens W.C., Schwacha M.G., Rue L.W., Bland K.I., Chaudry I.H. (2004). Estradiol’s effect on portal response to endothelin-1 after trauma-hemorrhage. J. Surg. Res..

[48] Gatson J.W., Maass D.L., Simpkins J.W., Idris A.H., Minei J.P., Wigginton J.G. (2009). Estrogen treatment following severe burn injury reduces brain inflammation and apoptotic signaling. J. Neuroinflammation.

[49] Mizushima Y., Wang P., Jarrar D., Cioffi W.G., Bland K.I., Chaudry I.H. (2000). Estradiol administration after trauma-hemorrhage improves cardiovascular and hepatocellular functions in male animals. Ann. Surg.

[50] Ye C., Qi M., Fan Q.W., Ito K., Akiyama S., Kasai Y., Matsuyama M., Muramatsu T., Kadomatsu K. (1999). Expression of midkine in the early stage of carcinogenesis in human colorectal cancer. Br. J. Cancer.

[51] Konishi N., Nakamura M., Nakaoka S., Hiasa Y., Cho M., Uemura H., Hirao Y., Muramatsu T., Kadomatsu K. (1999). Immunohistochemical analysis of midkine expression in human prostate carcinoma. Oncology.

[52] Sakitani H., Tsutsumi M., Kadomatsu K., Ikematsu S., Takahama M., Iki K., Tsujiuchi T., Muramatsu T., Sakuma S., Sakaki T. (1999). Overexpression of midkine in lung tumors induced by N-nitrosobis(2-hydroxypropyl)amine in rats and its increase with progression. Carcinogenesis.

[53] Ikematsu S., Nakagawara A., Nakamura Y., Ohira M., Shinjo M., Kishida S., Kadomatsu K. (2008). Plasma midkine level is a prognostic factor for human neuroblastoma. Cancer Sci..

[54] Zhu W.W., Guo J.J., Guo L., Jia H.L., Zhu M., Zhang J.B., Loffredo C.A., Forgues M., Huang H., Xing X.J. (2013). Evaluation of midkine as a diagnostic serum biomarker in hepatocellular carcinoma. Clin. Cancer Res..

[55] Kosugi T., Yuzawa Y., Sato W., Arata-Kawai H., Suzuki N., Kato N., Matsuo S., Kadomatsu K. (2007). Midkine is involved in tubulointerstitial inflammation associated with diabetic nephropathy. Lab. Investig..

[56] Horiba M., Kadomatsu K., Nakamura E., Muramatsu H., Ikematsu S., Sakuma S., Hayashi K., Yuzawa Y., Matsuo S., Kuzuya M. (2000). Neointima formation in a restenosis model is suppressed in midkine-deficient mice. J. Clin. Investig..

[57] Takada T., Toriyama K., Muramatsu H., Song X.J., Torii S., Muramatsu T. (1997). Midkine, a retinoic acid-inducible heparin-binding cytokine in inflammatory responses: Chemotactic activity to neutrophils and association with inflammatory synovitis. J. Biochem..

[58] Krzystek-Korpacka M., Mierzchala M., Neubauer K., Durek G., Gamian A. (2011). Midkine, a multifunctional cytokine, in patients with severe sepsis and septic shock: A pilot study. Shock.

[59] Diamond-Stanic M.K., Romero-Aleshire M.J., Hoyer P.B., Greer K., Hoying J.B., Brooks H.L. (2011). Midkine, a heparin-binding protein, is increased in the diabetic mouse kidney postmenopause. Am. J. Physiol. Renal. Physiol..

[60] Pountos I., Georgouli T., Giannoudis P.V. (2008). The effect of autologous serum obtained after fracture on the proliferation and osteogenic differentiation of mesenchymal stem cells. Cell. Mol. Biol..

[61] Kaspar D., Neidlinger-Wilke C., Holbein O., Claes L., Ignatius A. (2003). Mitogens are increased in the systemic circulation during bone callus healing. J. Orthop. Res..

[62] Pountos I., Georgouli T., Henshaw K., Bird H., Giannoudis P.V. (2013). Release of growth factors and the effect of age, sex, and severity of injury after long bone fracture. A preliminary report. Acta. Orthop..

[63] Haffner-Luntzer M., Kovtun A., Fischer V., Prystaz K., Hainzl A., Kroeger C.M., Krikki I., Brinker T.J., Ignatius A., Gatzka M. (2018). Loss of p53 compensates osteopenia in murine Mysm1 deficiency. FASEB J..

[64] Ramakers C., Ruijter J.M., Deprez R.H., Moorman A.F. (2003). Assumption-free analysis of quantitative real-time polymerase chain reaction (PCR) data. Neurosci. Lett..

